# Dual-layer spectral detector CT for improved assessment of right adrenal vein in patients with primary aldosteronism

**DOI:** 10.1007/s00261-025-05165-7

**Published:** 2025-08-18

**Authors:** Ryuya Yoshida, Yasunori Nagayama, Takumi Esaki, Taiga Matsumoto, Yoshitaka Tamura, Soichiro Ishiuchi, Taihei Inoue, Daisuke Sakabe, Yasuaki Igarashi, Masafumi Kidoh, Seitaro Oda, Takeshi Nakaura, Hiro Kiyosue, Toshinori Hirai

**Affiliations:** 1 Department of Diagnostic Radiology, Kumamoto University, Kumamoto, Japan; 2 Central Radiology, Kumamoto University Hospital, Kumamoto, Japan; 3 Department of Radiology, Nippon Medical School Hospital, Tokyo, Japan; 4 Department of Radiology, National Defense Medical College, Saitama, Japan

**Keywords:** Adrenal gland, Primary aldosteronism, Adrenal venous sampling, Dual-energy CT, Virtual monoenergetic image

## Abstract

**Purpose:**

To investigate whether virtual-monoenergetic images (VMI) from dual-layer spectral-detector computed tomography (DLCT) improve right adrenal vein (RAV) assessment before adrenal venous sampling (AVS), and portal-venous-phase (PVP) images alone can replace conventional multiphase CT combining late-arterial phase (LAP) and PVP.

**Methods:**

Sixty-three patients with primary aldosteronism who underwent LAP and PVP DLCT before AVS were retrospectively analyzed. Conventional polyenergetic-images (PEI) and VMI at 40–70 keV (VMI_40–70_) were reconstructed. Image noise and contrast-to-noise ratio (CNR) of RAV were quantified. VMI with the highest CNR was predefined as the best-VMI. Two radiologists evaluated four image series (PEI and best-VMI at LAP and PVP) separately for RAV visibility, accessory hepatic vein (AHV) assessability, and image noise using five-point scales (1 = worst, 5 = best). RAV detection and AHV assessability rates were compared among image series.

**Results:**

In both enhancement phases, VMI_40–70_ showed lower image noise and VMI_40–60_ higher CNR than PEI (all *p* < 0.05), with VMI_40_ providing the highest CNR and being designated as the best VMI. Compared to PEI at LAP, CNR was lower with PEI at PVP but higher with VMI_40_ at PVP (both, *p* < 0.001). In subjective analysis, VMI_40_ showed better scores than PEI for image noise (LAP: 4.0 ± 0.5 vs. 2.4 ± 0.6; PVP: 4.0 ± 0.6 vs. 2.5 ± 0.5) and RAV visibility (LAP: 4.5 ± 0.8 vs. 3.5 ± 0.8; PVP: 3.7 ± 1.0 vs. 2.6 ± 0.9) (all *p* < 0.001). With PEI, the RAV detection rate was lower at PVP than at LAP (54.8% vs. 89.7%, *p* < 0.001). Use of VMI_40_ at PVP achieved the same detection rate as PEI at LAP (89.7%, *p* = 1.0) and higher AHV assessability rate than PEI at PVP (96.8% vs. 80.2%, *p* < 0.001).

**Conclusion:**

DLCT-VMI improved RAV assessments before AVS. Using VMI_40_ for PVP provided RAV depiction equivalent to PEI at LAP and superior AHV assessability to PEI at PVP, suggesting its potential as a surrogate for dedicated multiphase CT.

## 1. Introduction

Primary aldosteronism (PA) is the most common cause of secondary hypertension, with a reported prevalence of 1.4–10% among hypertensive patients [1]. PA causes irreversible organ damage and increases the risk of cardiovascular disease, making early diagnosis and treatment essential [1, 2]. Unilateral PA is primarily treated with surgical resection, whereas bilateral PA is managed with medications [3]. Adrenal venous sampling (AVS) is indispensable for determining aldosterone lateralization and guiding appropriate treatment strategies [4–6].

The most technically challenging aspect of AVS is the selective catheterization of right adrenal vein (RAV) due to its small size, anatomical variations, and complex location, which affect procedural success rates [4]. RAV typically drains directly into the inferior vena cava (IVC), but a common trunk with accessory hepatic vein (AHV) is also frequently observed. Therefore, superselective cannulation is required to prevent contamination with hepatic blood [5, 6]. Moreover, small AHV may mimic RAV during venography, leading to miscatheterization [6]. Accurate preprocedural assessment of the anatomical relationship between RAV and AHV is crucial for improving technical success rates, reducing procedure time, and minimizing radiation exposure.

Multiphase contrast-enhanced computed tomography (CT) enables the precise assessment of RAV anatomy and is recommended before AVS [6–9]. The late arterial phase (LAP) provides the best visualization of RAV, while portal venous phase (PVP) best depicts its relationship with AHV [6, 7, 10, 11]. However, multiphase scanning involves relatively high radiation exposure, raising concerns about the potential adverse effects. Furthermore, if only routine PVP images are available (e.g., in cases where adrenal incidentalomas are later diagnosed as PA), an additional LAP scan would be needed for better RAV visualization, resulting in contrast media re-administration, increased radiation exposure, and higher medical costs. These limitations underscore the need for an optimized imaging strategy that enables comprehensive RAV and AHV assessment within a single-phase PVP acquisition.

Dual-energy CT (DECT) enables the reconstruction of virtual monoenergetic images (VMI) at low keV levels, which increases iodine attenuation and enhances RAV contrast because of its proximity to the iodine K-edge [12, 13]. An inherent limitation of low-keV VMI is increased image noise, which may impair the technique's utility for visualizing low-contrast objects such as abdominal parenchymal lesions and suboptimally opacified RAV on routine PVP CT [12–15]. In addition, tube-based DECT systems require prospective selection of dual-energy mode, also limiting their applicability for incidental lesions. Among DECT systems, dual-layer spectral-detector CT (DLCT) acquires fully coherent dual-energy data using a two-layer detector, enabling basis decomposition in the projection domain without spatial or temporal interpolation. This configuration reduces noise variation across the energy spectrum, maximizing the utility of VMI at the lowest energy levels [16–21]. DLCT also allows retrospective VMI reconstruction for all acquisitions, suggesting that even incidental adrenal lesions—up to 5% of which are PA [22]— detected on single-phase PVP CT could benefit from enhanced RAV conspicuity. Considering these advantages, we hypothesized that low-keV VMI from DLCT could enhance RAV assessment before AVS and that single-phase PVP alone could replace dedicated multiphase CT. To our knowledge, no prior studies have investigated this hypothesis.

Therefore, this study aimed to evaluate the effectiveness of DLCT-VMI in PA patients before AVS, specifically to determine whether low-keV VMI in PVP can substitute for multiphase CT in the accurate assessment of RAV.

## Material and methods

This retrospective study received institutional review board approval; the requirement for written informed consent was waived.

### Patients

By searching our radiological database, we identified patients with clinical suspicion of PA who underwent multiphase CT prior to AVS on a DLCT scanner (IQon Spectral CT; Philips Healthcare, Netherlands) between July 2017 and October 2023. Patients were excluded if they underwent contrast-enhanced CT with protocols differing from our standardized contrast medium protocol (detailed in the Image acquisition section), if their spectral image data were unavailable, or if severe artifacts precluded accurate image quality evaluation.

### Image acquisition

All scanning was performed with the following acquisition parameters: tube voltage, 120 kVp; helical pitch, 0.798; detector collimation, 64 × 0.625; rotation time, 0.5 s. Tube current was modulated by automatic exposure control (Dose Right Index, 22; Philips Healthcare, Best, the Netherlands). A 600 mgI/kg dose of contrast medium (Iopamidol [Bayer Yakuhin, Japan], Iohexol [GE Healthcare Pharma, Japan], or Iomeprol [Bracco Japan, Japan]) was injected via antecubital vein over 30 s, followed by a 40 mL saline flush at the same injection rate. The image acquisition timing was determined using the bolus tracking technique. LAP and PVP scanning started 23 and 40 s, respectively, after the bolus tracking trigger of 150 HU at the abdominal aorta. The same raw data from each enhancement phase were used to reconstruct conventional 120 kVp polyenergetic images (PEI) and VMI at 40–70 keV (VMI_40–70_) in 10 keV increments on a post-processing workstation (IntelliSpace Portal, Philips Healthcare, Netherlands). A hybrid iterative reconstruction algorithm (iDose^4^ level 3, Philips Healthcare, Netherlands) was used for PEI. VMI was reconstructed using a dedicated spectral reconstruction algorithm (Spectral level 3, Philips Healthcare, Netherlands). Axial, sagittal and coronal images were reconstructed at 1.0 mm section thickness with 1.0 mm increments. Radiation dose was estimated using size-specific dose estimates (SSDE) with the volume CT dose index (CTDIvol) and patient effective diameter quantified on axial images at the right adrenal level:

$$ {\mathrm{SSDE}} = 3.70 \times {\mathrm{e}}^{{ - (0.0367 \, \times {\text{ effective}}\;{\mathrm{diameter}})}} \times {\mathrm{CTDIvol}} $$

Since this study compared RAV conspicuity between LAP and PVP, SSDE values were compared between both phases to evaluate whether image comparisons were conducted under identical radiation dose conditions.

### Objective analysis

Objective quantification was performed by a board-certified radiologist and a radiological technologist with more than 10 years of experience in abdominal imaging and the scientific quantitative image evaluation by consensus. For each enhancement phase, CT attenuation of RAV (HU_RAV_), adrenal gland (AG, HU_AG_), and spinal erector muscle (HU_muscle_) was measured by placing circular regions of interest (ROIs). ROIs for the RAV and AG were placed as large as possible while carefully avoiding inclusion of adjacent structures such as fat, typically measuring approximately 1 mm^2^ and 3 mm^2^, respectively. For the spinal erector muscle, a standardized ROI of approximately 100 mm^2^ was placed in a homogeneous region. Each ROI was placed by consensus of the two operators. Image noise was defined as the standard deviation (SD) of HU_muscle_ following prior studies [12–15, 20, 21, 23, 24]. Muscle was chosen because it provides a large, homogeneous anatomical region suitable for reliable and reproducible noise quantification. In contrast, the fat surrounding the RAV and AG was not used due to insufficient area for consistent measurements in some patients. The SD of HU within RAV or AG were also not used because these structures are too small and contain a limited number of pixels, usually exhibiting inhomogeneous enhancement affected by contrast dynamics, making them unsuitable for noise analysis. The signal-to-noise ratio (SNR) of RAV and AG was calculated by dividing HU_RAV_ and HU_AG_ by image noise, respectively. The contrast-to-noise ratio (CNR) of RAV and AG was calculated with the following equations [12, 13, 23]:

$$ {\mathrm{CNR}}_{{{\mathrm{RAV}}}} = \, \left( {{\mathrm{HU}}_{{{\mathrm{RAV}}}} {-}{\text{ HU}}_{{{\mathrm{muscle}}}} } \right)/{\mathrm{image}}\;{\mathrm{noise}} $$

$$ {\mathrm{CNR}}_{{{\mathrm{AG}}}} = \, \left( {{\mathrm{HU}}_{{{\mathrm{AG}}}} - {\text{ HU}}_{{{\mathrm{muscle}}}} } \right)/{\mathrm{image}}\;{\mathrm{noise}} $$

Among VMIs, the monoenergetic level yielding the highest CNR_RAV_ at each enhancement phase was predefined as the best energy. Subjective analysis was subsequently performed using this VMI series, as prior studies have demonstrated that images with the highest CNR provide the greatest object detectability and are most relevant for evaluating the added value of VMI [12, 13, 21, 24].

### Subjective analysis

Two board-certified radiologists with 4 and 12 years of experience in abdominal CT, not involving the objective analysis, independently evaluated the image quality of PEI and VMI at the best energy determined in objective image analysis. Each image series was presented to the readers using a clinical PACS viewer (ShadeQuest/ViewR-DG, FUJIFILM Medical Solutions Corporation, Japan), with patient information and acquisition parameters anonymized. For each patient, four image series (PEI and best VMI at LAP and PVP, a total of 63 × 4 = 252 image sets) were assessed separately in random order. Each image was initially displayed with a soft-tissue window (window level/width: 50/350 HU), but the readers optimized the settings to appropriately delineate RAV, as low-keV images require specific adjustments to adequately depict the region of interest and to avoid misinterpretation [25–28]. The subjective evaluation employed five-point scales for (a) RAV visibility [29, 30], (b) AHV assessability, (c) AG delineation, and (d) image noise [15, 31] (Table 1). The scoring criteria for RAV visibility and image noise were adopted from prior studies [15, 29–31], whereas those for AHV assessability and AG delineation were developed specifically for this study due to the lack of relevant prior research. Following the evaluation of inter-observer agreement, the average scores from the two readers were statistically compared among four image series.

**Table 1:** Subjective scoring scale

Score	RAV visibility	AHV assessability	Adrenal gland delineation	Image noise
1	Not visible	Hepatic vein not depicted and relationship between RAV or IVC and AHV not assessable	Poor	Undiagnostic
2	Equivocal, low confidence	Poor depiction and equivocally assessable	Suboptimal	Suboptimal
3	Moderate conspicuity, adequate confidence	Moderate depiction, adequately assessable	Average	Moderate
4	Good conspicuity, improved confidence	Good depiction, improved confidence	Good	Mild
5	Excellent conspicuity, the highest confidence	Excellent depiction, the highest confidence	Excellent	Absent

RAV: right adrenal vein, AHV: accessory hepatic vein

### RAV detection and AHV assessable rates

An RAV visibility score ≥ 3 and an AHV assessability score ≥ 3 in the subjective analysis were considered detectable and assessable image series, respectively. RAV detection and AHV assessability rates were calculated for each image series as follows:

$$ \begin{aligned} & {\mathrm{RAV}}\,{\mathrm{detection}}\,{\mathrm{rate}}\left( \% \right) \\ & \quad = \left( {{\mathrm{Number}}\,{\mathrm{of}}\,{\mathrm{detectable}}\,{\mathrm{cases/Total}}\,{\mathrm{number}}\,{\mathrm{of}}\,{\mathrm{cases}}} \right) \times 100 \\ \\ \\ \end{aligned} $$

$$ \begin{aligned} & {\mathrm{AHV}}\,{\mathrm{assessable}}\,{\mathrm{rate}}\left( \% \right) \\ & \quad = \left( {{\mathrm{Number}}\,{\mathrm{of}}\,{\mathrm{assessable}}\,{\mathrm{cases/Total}}\,{\mathrm{number}}\,{\mathrm{of}}\,{\mathrm{cases}}} \right) \times 100 \\ \\ \\ \end{aligned} $$

RAV detection and AHV assessability rates for the four image series (PEI and the best VMI at both LAP and PVP) were calculated separately for each reader. Additionally, overall detection and assessability rates were determined by combining the results from both readers, summing the number of detected or assessable cases (numerators) and the total number of evaluated cases (denominators) for each series.

### AVS procedure

AVS was performed using a unilateral femoral vein approach with a 4-F sheath. A 4-F catheters and 2.2-F microcatheter were used to sample blood from RAV, proximal and distal left adrenal veins, and infrarenal IVC. The correct position of the catheter in the RAV was confirmed using interventional-CT imaging during the procedure. Successful catheterization was defined as a selectivity index of ≥ 5, calculated as the cortisol ratio between RAV and IVC [6].

### Statistical analysis

MedCalc statistical software and DATAtab (https://datatab.net) were used for statistical analyses. All variables are expressed as means ± SDs. After confirming that all quantitative variables were normally distributed with the Kolmogorov–Smirnov test, a paired t-test was used for the comparison of SSDE between LAP and PVP, and ANOVA with repeated measures with post-hoc Bonferroni correction was applied to compare objective variables. Subjective scores were compared using Dunn’s test with post hoc Bonferroni correction. RAV detectable rate and AHV assessable rate were compared among the four image series (PEI at LAP and PVP, and best VMI at LAP and PVP) using the McNemar test with Holm correction. Interobserver variabilities in subjective quality scores were evaluated using the Kappa coefficient, with the following definitions: < 0.20, poor; 0.21–0.40, fair; 0.41–0.60, moderate; 0.61–0.80, substantial; and 0.81–1.00, excellent [32]. Differences of *p* < 0.05 were considered statistically significant.

## 3. Results

### Patient demographics and radiation dose

**Fig. 1:**
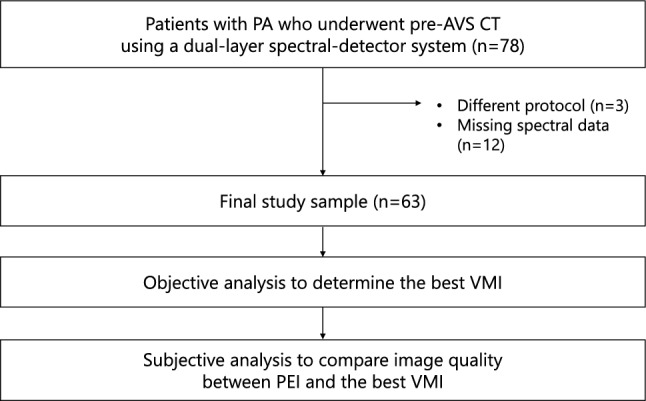
Study flowchart

A total of 78 patients met the inclusion criteria. Among these, 3 patients who underwent different CT protocols and 12 patients with missing spectral data were excluded. Consequently, 63 patients (28 males, 35 females; mean age: 53.0 ± 11.4 years) constituted the final study sample (Fig. 1 and Table 2). There was no significant difference in the SSDE between LAP and PVP (18.6 ± 4.1 vs. 18.6 ± 3.8 mGy, *p* = 1.0), indicating that comparisons of image quality between the two phases were performed under identical radiation exposure settings.

**Table 2:** Patient characteristics and radiation dose

Characteristic	N (%) or mean ± standard deviation
Age (years)	53.0 ± 11.4
Male/female	28 (44.4)/35 (55.6)
Body mass index (kg/m^2^)	25.4 ± 6.1
Effective body diameter (cm)	25.5 ± 3.1
AVS procedure	
Success/ non-success	62 (98.4)/ 1 (1.6)
Laterality of hormone excess*	
Bilateral/unilateral	33 (53.2)/29 (46.8)
Common trunk of RAV with AHV	
Presence/absence	12 (19.0)/51 (81.0)
Radiation dose	
CTDIvol during LAP (mGy)	13.1 ± 4.2
CTDIvol during PVP (mGy)	13.0 ± 3.9
SSDE during LAP (mGy)	18.6 ± 4.1
SSDE during PVP (mGy)	18.5 ± 3.8

AVS: adrenal venous sampling, CTDIvol: volume CT dose index, SSDE: size-specific dose estimate; RAV: right adrenal vein, AHV: accessory hepatic vein; LAP: late arterial phase, PVP: portal venous phase

*Data only for cases with successful AVS

### Objective analysis

**Fig. 2:**
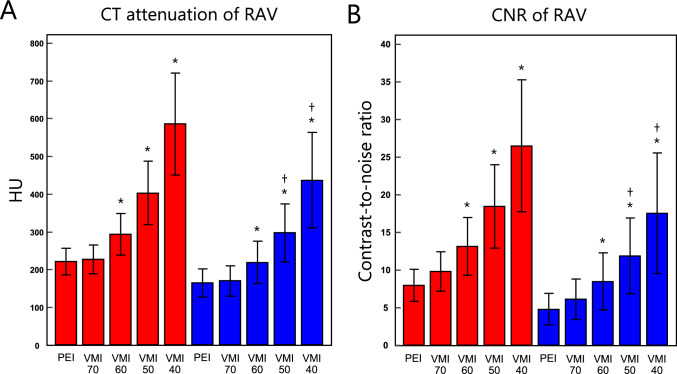
Bar chart showing mean CT attenuation and contrast-to-noise ratio (CNR) of the right adrenal vein (RAV) in the late arterial phase (red boxes) and portal venous phase (blue boxes) in polyenergetic images (PEI) and virtual monoenergetic images at 40–70 keV (VMI 40–70). The error bars represent the standard deviations. *VMI > PEI at each enhancement phase (*p* < 0.05). †VMI at PVP > PEI at LAP (*p* < 0.05)

For each enhancement phase, the image noise of VMI remained nearly consistent across the energy range (differences of ≤ 2.1 HU) and was significantly lower than that of PEI (all, *p* < 0.05). In VMI, the CT attenuation, SNR, and CNR of RAV and AG gradually increased as the energy decreased, reaching a maximum at VMI_40_. For each phase, CT attenuation, SNR, and CNR of RAV and AG of VMI_40–60_ were significantly higher than those of conventional PEI (all, *p* < 0.001). When PEI at LAP was used as a reference image for inter-phase comparisons, CT attenuation, SNR, and CNR of RAV in PVP were significantly lower when using PEI reconstruction (*p* < 0.001) but significantly higher when using VMI_40–50_ (*p* < 0.001). The full results of the objective analysis are shown in Fig. 2 and Table 3.

**Table 3:** Objective analysis

	Conventional PEI	VMI 70 keV	VMI 60 keV	VMI 50 keV	VMI 40 keV
Late arterial phase					
Image noise (HU)	21.1 ± 4.0	†17.7 ± 3.4	†18.5 ± 5.2	†18.8 ± 3.9	†20.0 ± 5.0
CT attenuation (HU)					
RAV	221.9 ± 34.8	227.2 ± 37.9	*294.2 ± 55.6	*403.1 ± 83.9	*586.0 ± 134.5
Adrenal gland	147.4 ± 23.6	153.0 ± 24.2	*201.4 ± 33.3	*279.9 ± 46.7	*417.7 ± 73.9
SNR					
RAV	10.8 ± 2.4	*13.2 ± 2.9	*16.6 ± 4.2	*22.2 ± 5.9	*30.6 ± 9.1
Adrenal gland	7.2 ± 1.7	*8.9 ± 2.1	*11.4 ± 2.8	*15.5 ± 3.7	*21.9 ± 6.1
CNR					
RAV	8.0 ± 2.1	9.8 ± 2.6	*13.1 ± 3.8	*18.4 ± 5.5	*26.4 ± 8.7
Adrenal gland	4.4 ± 1.5	*5.6 ± 1.9	*8.0 ± 2.6	*11.7 ± 3.5	*17.8 ± 5.8
Portal venous phase					
Image noise (HU)	21.8 ± 3.8	†18.1 ± 3.1	†18.4 ± 3.3	†19.1 ± 3.6	†20.4 ± 4.5
CT attenuation (HU)					
RAV	164.7 ± 37.7	170.3 ± 40.7	*219.6 ± 56.3	*297.8 ± 77.2	*437.4 ± 126.8
Adrenal gland	109.9 ± 20.1	115.2 ± 19.4	*147.2 ± 24.2	*201.6 ± 33.2	*292.2 ± 48.9
SNR					
RAV	7.8 ± 2.3	9.7 ± 3.0	*12.3 ± 4.1	*16.1 ± 5.3	*22.4 ± 8.4
Adrenal gland	5.2 ± 1.4	*6.5 ± 1.7	*8.3 ± 2.1	*10.9 ± 2.8	*14.9 ± 3.9
CNR					
RAV	4.8 ± 2.1	6.1 ± 2.7	*8.5 ± 3.8	*11.9 ± 5.0	*17.5 ± 8.0
Adrenal gland	2.3 ± 1.2	*3.0 ± 1.4	*4.5 ± 1.8	*6.7 ± 2.5	*10.1 ± 3.5

Data shown are mean ± standard deviation. *VMI > PEI at each enhancement phase (*p* < 0.05). †VMI < PEI at each enhancement phase (*p* < 0.05). **Bold numbers** indicate comparisons in which values of the portal venous phase are significantly higher than those of PEI at the late arterial phase (*p* < 0.05)

PEI: polyenergetic image, VMI: virtual monoenergetic image, RAV: right adrenal vein, SNR: signal-to-noise ratio, CNR: contrast-to-noise ratio

### Subjective analysis

Based on the results of objective analysis, subjective analysis was conducted using PEI and VMI_40_. For both PEI and VMI_40_, images obtained during LAP demonstrated higher subjective scores for RAV visualization and AG delineation, but lower AHV assessability scores compared to those obtained during PVP (all, *p* < 0.001). When compared within the same enhancement phase, VMI_40_ provided significantly better subjective scores than PEI across all evaluation criteria (*p* < 0.001), except for the AHV assessability score in LAP (*p* = 0.177). When compared to PEI at LAP, VMI_40_ at PVP yielded a significantly higher RAV visualization score (*p* = 0.021). There was moderate to substantial inter-reader agreement (kappa = 0.507–0.723). Table 4 summarizes the results of subjective analysis.

**Table 4:** Subjective analysis

Parameters	Image series				*P* values for pairwise comparisons						kappa
	PEI at LAP	PEI at PVP	VMI_40_ at LAP	VMI_40_ at PVP	PEI at LAP vs PEI at PVP	PEI at LAP vs VMI_40_ at LAP	PEI at LAP vs VMI_40_ at PVP	PEI at PVP vs VMI_40_ at LAP	PEI at PVP vs VMI_40_ at PVP	VMI_40_ at LAP vs VMI_40_ at PVP	
RAV visibility	3.5 ± 0.8	2.6 ± 0.9	4.5 ± 0.8	3.7 ± 1.0	0.001	< 0.001	0.021	< 0.001	< 0.001	0.019	0.723
AHV assessability	1.2 ± 0.5	3.5 ± 0.7	1.7 ± 0.7	4.6 ± 0.7	< 0.001	0.177	< 0.001	< 0.001	< 0.001	< 0.001	0.507
Image noise	2.4 ± 0.6	2.5 ± 0.5	4.0 ± 0.5	4.0 ± 0.6	1.0	< 0.001	< 0.001	< 0.001	< 0.001	1.0	0.595
Adrenal grand delineation	3.3 ± 0.4	2.2 ± 0.4	4.8 ± 0.4	3.7 ± 0.5	< 0.001	< 0.001	0.032	< 0.001	< 0.001	< 0.001	0.614

Data shown are mean ± standard deviation. PEI: polyenergetic image, VMI_40_: virtual monoenergetic image at 40 keV, RAV: right adrenal vein, AHV: accessory hepatic vein, LAP: late arterial phase, PVP: portal venous phase. The averaged scores from two readers were analyzed

### RAV detection and AHV assessable rates

**Fig. 3:**
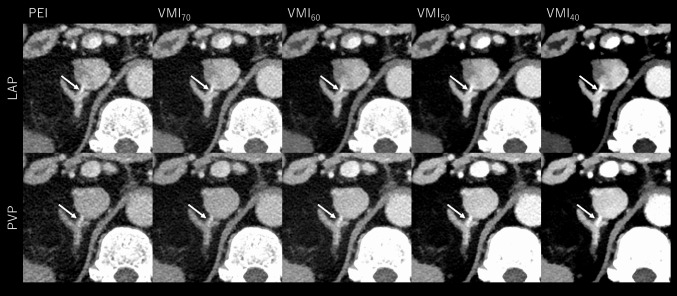
CT images of a 65-year-old male with PA (BMI: 29.1 kg/m^2^). The upper and lower rows display late arterial phase (LAP) and portal venous phase (PVP) images, respectively, reconstructed with polyenergetic images (PEI) and virtual monoenergetic images at 40–70 keV (VMI_40-70_). The window display is optimized for appropriate RAV assessment for each image series. In VMI, the contrast and depiction of the right adrenal vein (RAV, arrows) directly draining into inferior vena cava were maximized at VMI_40_. The perceived image noise in VMIs decreases as energy levels decrease, as a wider window display was used for lower-energy images to optimize image interpretation

**Fig. 4:**
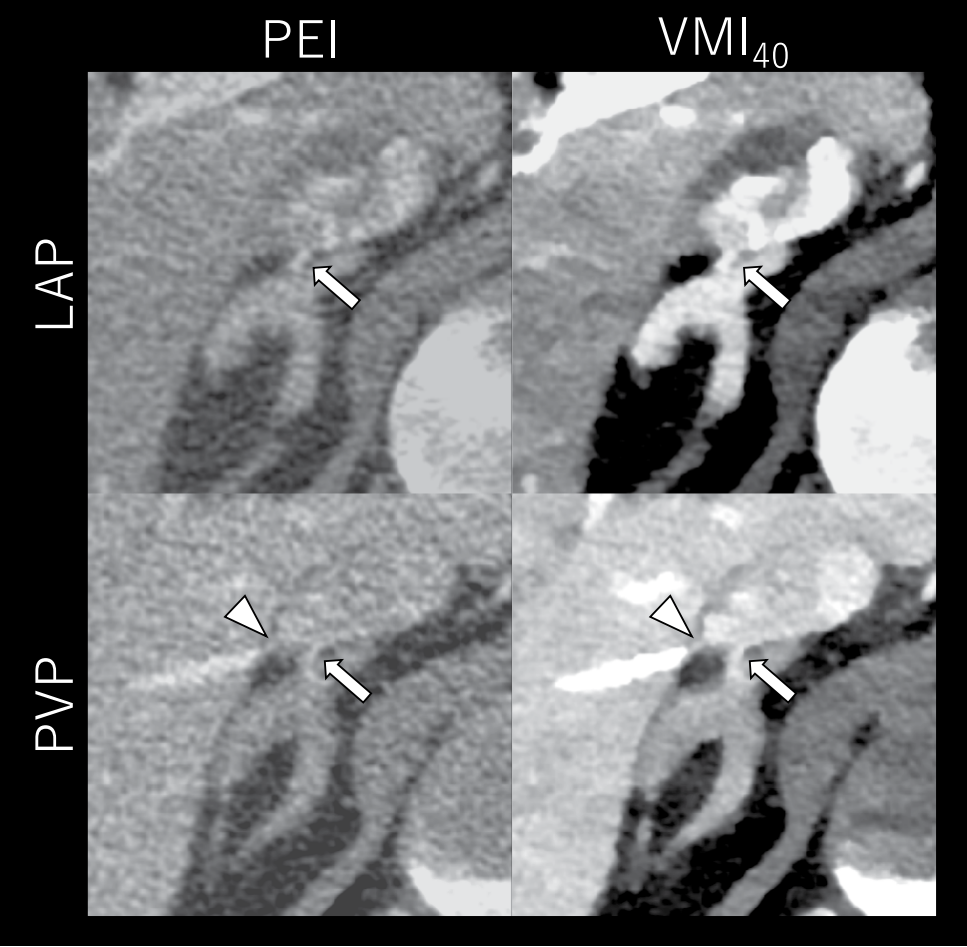
CT images of a 44-year-old male with bilateral PA (BMI: 24.4 kg/m^2^). The upper and lower rows display late arterial phase (LAP) and portal venous phase (PVP) images, respectively, reconstructed with polyenergetic images (PEI) and virtual monoenergetic images at 40 keV (VMI_40_). The window display is optimized for each image series.VMI_40_ at PVP most clearly delineated the relationship between the RAV (arrows) and accessory hepatic vein (arrowheads), both independently draining into the inferior vena cava, while also providing adequate RAV visibility

**Fig. 5:**
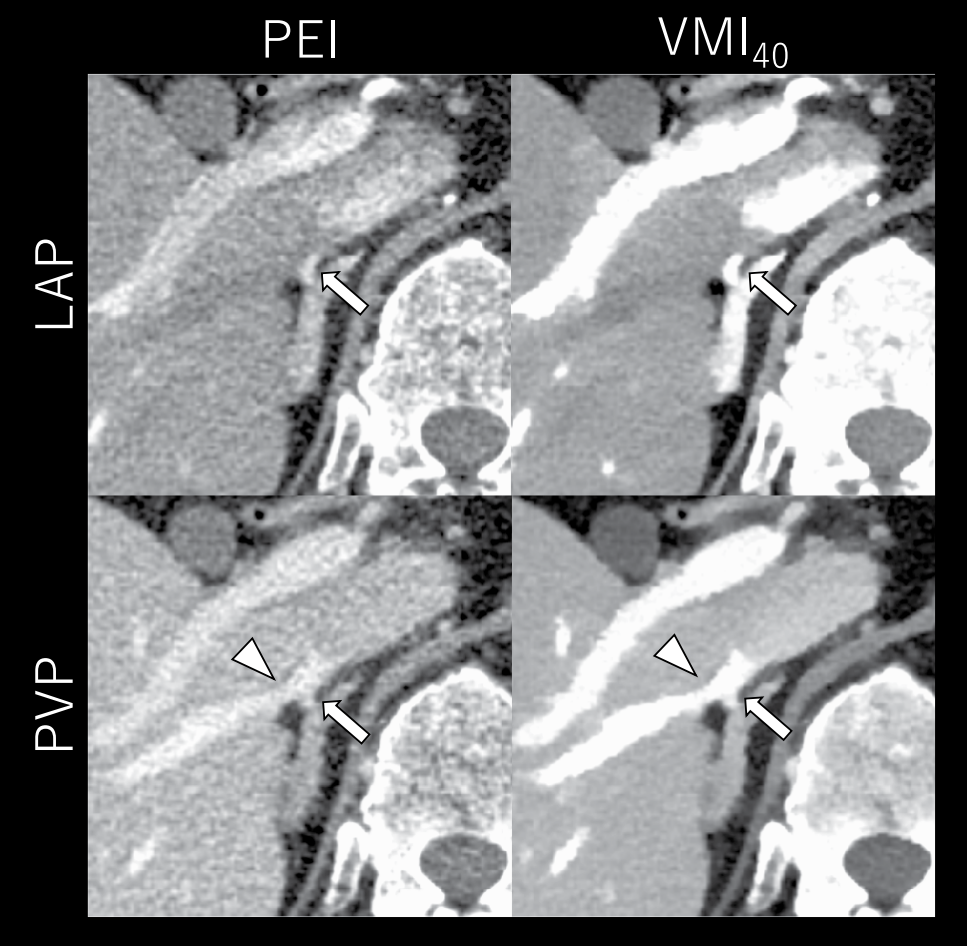
CT images of a 66-year-old male with primary aldosteronism (BMI: 26.5 kg/m^2^). The upper and lower rows display late arterial phase (LAP) and portal venous phase (PVP) images, respectively, reconstructed with polyenergetic images (PEI, left column) and virtual monoenergetic images at 40 keV (VMI_40_, right column). The window display is optimized for each image series. VMI_40_ at PVP provided a clearer depiction of RAV (arrows) than PEI at LAP and most effectively visualized its relationship with accessory hepatic vein (arrowheads), both draining into the inferior vena cava after forming a common trunk

The results of the RAV detection and AHV assessable rates are summarized in Table 5. Combining the results from both readers, the RAV detection rate was significantly higher in LAP than in PVP when using conventional PEI (89.7% vs. 54.8%, *p* < 0.001). In PVP, RAV detection rate significantly improved with VMI_40_ compared to PEI (89.7%, *p* < 0.001), achieving the same detection rate as PEI at LAP (*p* = 1.00). The AHV assessable rate was significantly higher in PVP than in LAP with PEI (80.2% vs. 2.4%, *p* < 0.001) and further improved with VMI_40_ in PVP (96.8%, *p* < 0.001). Representative cases are shown in Figs. 3, 4, 5.

**Table 5:** RAV detectability and AHV assessable rate

Parameter	LAP		PVP		P values for pairwise comparisons					
	PEI	VMI_40_	PEI	VMI_40_	PEI LAP vs VMI_40_ LAP	PEI LAP vs PEI PVP	PEI LAP vs VMI_40_ PVP	VMI_40_ LAP vs PEI PVP	VMI_40_ LAP vs VMI_40_ PVP	PEI PVP vs VMI_40_ PVP
Reader 1										
RAV detection rate	90.5% (57/63)	98.4% (62/63)	55.6% (35/63)	85.7% (54/63)	0.125	< 0.001	0.370	< 0.001	0.023	< 0.001
AHV assessable rate	3.2% (2/63)	11.1% (7/63)	85.7% (54/63)	96.8% (61/63)	0.063	< 0.001	< 0.001	< 0.001	< 0.001	0.031
Reader 2										
RAV detection rate	88.9% (56/63)	98.4% (62/63)	54.0% (34/63)	93.7% (59/63)	0.093	< 0.001	0.453	< 0.001	0.750	< 0.001
AHV assessable rate	1.6% (1/63)	11.1% (7/63)	74.6% (47/63)	96.8% (61/63)	0.031	< 0.001	< 0.001	< 0.001	< 0.001	< 0.001
Reader 1 + 2*										
RAV detection rate	89.7% (113/126)	98.4% (124/126)	54.8% (69/126)	89.7% (113/126)	0.004	< 0.001	1.0	< 0.001	0.007	0.010
AHV assessable rate	2.4% (3/126)	11.1% (14/126)	80.2% (101/126)	96.8% (122/126)	< 0.001	< 0.001	< 0.001	< 0.001	< 0.001	< 0.001

PEI: polyenergetic image, VMI_40_: virtual monoenergetic image at 40 keV, RAV: right adrenal vein, AHV: accessory hepatic vein, LAP: late arterial phase, PVP: portal venous phase. RAV visibility score ≥ 3 indicates that RAV is detectable. AHV assessability score ≥ 3 indicates that the relationship between RAV, AHV, and IVC, and presence or absence of common trunk is assessable. *The combined results were calculated by summing the numerators and denominators from both readers

## 4. Discussions

This study demonstrated that DLCT-VMI significantly enhances RAV visualization in PA patients undergoing pre-AVS CT compared to conventional 120-kVp PEI. In both LAP and PVP, VMI_40_ maximized RAV contrast while maintaining lower image noise than PEI. Although PEI provided better RAV visualization in LAP than in PVP, using VMI_40_ for PVP achieved comparable RAV detectability to PEI at LAP. For evaluating the RAV-AHV relationship, PVP was more effective than LAP, with the highest visual scores observed in PVP VMI_40_. These findings suggest that VMI_40_ on routine PVP CT provides comparable RAV visualization to PEI at LAP while enhancing the assessment of the RAV-AHV relationship compared to PEI at PVP, showing its potential as an alternative to dedicated multiphase CT.

Lowering X-ray energy enhances iodine contrast as it approaches the iodine K-edge (33.2 keV). Several studies on single-energy scanning have demonstrated the effectiveness of low tube voltage techniques for pre-AVS CT. Takahashi et al. reported that 80 kVp scanning (600 mgI/kg) resulted in 1.6-fold higher RAV attenuation compared to 120 kVp (331.0 ± 65.5 vs. 209.9 ± 45.1 HU in LAP), allowing a 40% reduction in contrast medium dose while maintaining comparable RAV visualization to the 120 kVp protocol [33]. Maruyama et al. similarly showed that 70 kVp scanning (510 mgI/kg) increased RAV attenuation by up to 1.9 times (248.6 ± 69.3 vs. 130 ± 42.7 HU in LAP, 226.6 ± 47.3 vs. 133.5 ± 35.8 HU in PVP) while reducing radiation exposure compared to the 120 kVp protocol [34]. In this study, VMI_40_ achieved even higher RAV attenuation (586.0 ± 134.5 and 437.4 ± 126.8 HU in LAP and PVP, respectively) due to its closer proximity to the iodine K-edge compared to polyenergetic 70–80 kVp.

Currently, few studies have explored the usefulness of DECT-derived VMI for pre-AVS CT. Nakayama et al., who evaluated 21 PA patients found that VMI_40_ from second-generation dual-source DECT improved RAV contrast, CNR, and visibility compared to 120 kVp equivalent images [12]. Using third-generation dual-source DECT, Wang et al. assessed VMI_40–80_ in 20 PA patients undergoing AVS and reported that RAV visibility was highest at VMI_40_ during LAP (20 s after the arterial phase) [13]. Although Wang et al. noted that image noise of VMI_40_ was approximately 2.5 times higher than that of VMI_70_ (31.6 ± 8.8 vs. 12.5 ± 3.2 HU), this study found that image noise of DLCT-VMI remained nearly constant across energy levels (differences were within 13%) and that VMI_40_ showed lower noise than PEI. The observed low noise properties of DLCT-derived VMIs relative to PEI are consistent with findings from previous studies using DLCT [16, 17, 20, 21, 31]. This favorable noise performance may be attributed to the use of projection-based material decomposition with perfectly matched dual-energy data and an anti-correlated noise reduction algorithm, which enables the suppression and cancellation of noise during VMI reconstruction. Additional denoising is achieved through projection-based iterative reconstruction algorithms specifically designed for spectral imaging. Moreover, the use of a wider window setting in VMI_40_ for optimal image interpretation further reduced perceptible subjective noise, resulting in an even noiseless appearance [24–28, 31]. Given that image noise can impair RAV visibility [33, 35], the minimal increase in image noise combined with the substantial enhancement of iodine contrast in DLCT-VMI_40_ contributed to the improved RAV visibility and detection rates, especially in PVP CT where RAV opacification is typically suboptimal compared to LAP.

Enhancing RAV visibility and accurately recognizing its relationship with IVC and AHV are crucial for successful AVS procedure. A common trunk of RAV and AHV has been reported in 8–20% of cases [5, 7, 36] and was observed in 19% in this study. Consistent with previous reports, RAV detection and AHV assessability were better in LAP and PVP, respectively [6, 7, 10, 11], confirming that LAP and PVP are optimal timing for RAV and AHV evaluation, respectively. Although iodine concentration in RAV is maximized during LAP, hepatic veins are not opacified at this phase, resulting in inadequate AHV visibility even with VMI_40_. In contrast, during PVP, although iodine concentration in RAV is lower than in LAP, VMI_40_ provided sufficient RAV visibility, achieving a detection rate comparable to PEI at LAP. Moreover, the enhanced contrast of AHV with VMI_40_ improved the assessment of the RAV–AHV relationship, indicating that comprehensive RAV evaluation required for AVS can be achieved using VMI_40_ at PVP, potentially eliminating the need for dedicated multiphase CT in certain cases.

The study findings have several clinical implications. Utilizing VMI_40_ in dedicated multiphase CT allows for more confident RAV assessment compared to PEI, potentially improving procedural success rates and reducing procedure time. Furthermore, as demonstrated in other diagnostic tasks [37–39], contrast medium dose can be reduced while maintaining diagnostically adequate iodine contrast. Even in clinical settings where only routine PVP CT is available, VMI_40_ enables comprehensive RAV assessment and can serve as a surrogate for multiphase CT in most cases. This approach could eliminate the need for additional examinations, thereby reducing radiation exposure, avoiding contrast media reinjection, and lowering medical costs. In addition to low-keV VMI for improved assessment of the RAV, adrenal adenomas incidentally detected on PVP CT can be discriminated from non-adenomas using virtual non-contrast images, iodine maps, and fat-fraction maps derived from the same dual-energy dataset—even in the absence of true non-contrast or delayed-phase imaging [40–42]. This enables a comprehensive one-stop-shop approach for both vascular evaluation and lesion discrimination in a single scan.

This study had several limitations. First, it was a retrospective single-center study with a relatively small sample size, although it is the largest study on pre-AVS DECT to date. Second, only the standard imaging protocol at our institution was evaluated, limiting the generalizability of the results to different imaging acquisition timings or contrast injection protocols. In addition, this study utilized only a DLCT scanner, and the applicability of the findings to DECT systems from other vendors requires further investigation, given the considerable variation in VMI noise characteristics reported across different DECT platforms [18, 19]. Third, subjective scoring for AHV assessability and adrenal gland delineation was based on original criteria developed for this study due to the lack of validated methods in previous literature. Fourth, effect sizes to estimate clinical relevance for pairwise comparisons could not be reported, as the available statistical software does not support their calculation. Fifth, due to its retrospective design, this study did not directly assess the impact of VMI on AVS procedural success or reduction in procedure time. Previous studies have shown that preprocedural vascular imaging [10, 43, 44] and catheter position confirmation using intraprocedural CT imaging [23, 45] improve the procedural success rate of AVS. To expand and validate our findings into clinical practice, further large-scale prospective studies are required. Sixth, the ROI size for RAV attenuation measurements was limited by the small vessel caliber, making it sensitive to single-pixel noise and partial-volume effects. This limitation is inherent to the small size of the RAV and is common in similar quantitative image quality studies. To ensure measurement reliability, ROI placement was performed in consensus by two experienced operators in this study. Finally, although the image series were anonymized and blinded in the subjective analysis, the inherent differences in image appearance between PEI and VMI_40_, or between LAP and PVP, might have allowed readers to recognize the type of image.

In conclusion, DLCT VMI improved RAV visualization in PA patients eligible for AVS. Specifically, VMI_40_ at LAP provided optimal RAV depiction, while VMI_40_ at PVP was most suitable for assessing its anatomical relationship with AHV. Notably, VMI_40_ at PVP achieved RAV detection rates comparable to PEI at LAP, enabling comprehensive assessments for AVS and potentially serving as a viable alternative to conventional multiphase CT.

## Data Availability

No datasets were generated or analysed during the current study.

## Abbreviations

AG: Adrenal gland
AHV: Accessory hepatic vein
AVS: Adrenal venous sampling
CT: Computed tomography
DLCT: Dual-layer spectral detector CT
IVC: Inferior vena cava
LAP: Late arterial phase
PA: Primary aldosteronism
PEI: Polyenergetic image
PVP: Portal venous phase
RAV: Right adrenal vein
SSDE: Size-specific dose estimates
VMI: Virtual monoenergetic image
